# A Rare Case of Large Lateral Chest Wall Hibernoma

**DOI:** 10.7759/cureus.59943

**Published:** 2024-05-09

**Authors:** Lyubomir Gaydarski, Boycho Landzhov, Ivaylo Kamenov, Julian M Ananiev, Georgi P Georgiev

**Affiliations:** 1 Department of Anatomy, Histology and Embryology, Medical University of Sofia, Sofia, BGR; 2 Department of Orthopedics and Traumatology, University Hospital Queen Giovanna-ISUL, Sofia, BGR; 3 Department of General and Clinical Pathology, Forensic Medicine and Deontology, Trakia University, Stara Zagora, BGR; 4 Department of Orthopedics and Traumatology, University Hospital Queen Giovanna-ISUL, Medical University of Sofia, Sofia, BGR

**Keywords:** surgical treatment, tumor imaging, difficult diagnosis, chest wall tumour, hibernoma

## Abstract

Hibernomas, rare benign tumors originating from brown adipose tissue, pose diagnostic challenges due to their infrequent occurrence and slow growth. We present a case of a 38-year-old woman with a progressively enlarging mass in her right lateral chest wall, initially stable in size but growing during pregnancy and causing pain and functional impairment. Radiological evaluation, including x-ray and MRI, provided inconclusive results, necessitating a biopsy for a definitive diagnosis. Ultrasound-guided needle aspiration biopsy revealed typical histopathological features consistent with hibernoma. A subsequent total surgical excision with negative margins was performed. The patient achieved complete recovery without recurrence during two years of follow-up. This case underscores the importance of considering hibernoma in the differential diagnosis of adipose tissue tumors, particularly in atypical clinical presentations. Moreover, it highlights the challenges in diagnosing and managing hibernomas and emphasizes the role of MRI and biopsy in achieving accurate diagnosis and optimal treatment outcomes. Continued reporting of such cases is crucial for increasing awareness and improving the management of this rare tumor.

## Introduction

Hibernoma is a rare benign tumor originating from brown adipose tissue, constituting 1.6% of adipose tumors [1]. Brown fat, abundant in hibernating animals, serves metabolic and thermogenic roles. While present in human newborns, its prevalence diminishes significantly after the eighth week of life [2]. Furlong et al. [2] conducted a comprehensive study, reporting 170 cases of hibernoma, with 99 male and 71 female patients averaging 38 years of age (ranging from two to 75 years). These tumors are primarily located in the axillae, interscapular region, and groin, with less frequent occurrences in the jaw, neck, shoulder, back, chest, arm, and retroperitoneum [2]. Hibernomas are slow-growing tumors typically found painlessly in the subcutaneous regions of the thigh, upper trunk, and neck [3]. They often develop near the axial skeleton, where brown fat remnants from fetal development persist into adulthood [4]. Hibernomas usually appear as a soft, movable, lobulated mass enclosed in a capsule, with minimal invasion into surrounding tissues. They typically feel warm and measure around 5 to 10 cm in diameter, although more prominent cases have been reported [5]. Four histopathological isoforms of hibernoma exist typical hibernoma, lipoma-like hibernoma, myxoid variant, and spindle cell variant [6]. Unlike typical lipomas, hibernomas exhibit granular eosinophilic cytoplasm and multivacuolated cells with numerous lipid droplets. They also feature small, bland central nuclei with rare mitotic figures or atypia [3]. Lipoma-like hibernomas predominantly consist of mature univacuolated adipocytes with occasional eosinophilic granular cells and a few multivacuolated brown fat cells [6]. Various conditions, such as conventional lipoma, lipoblastoma, atypical lipomatous tumor, and others, should be considered in the differential diagnosis of hibernoma [7]. Overall, hibernoma is a rare pathological entity, with only around 200 cases published in the literature [4]. Moreover, sometimes, this tumor poses difficulties in making the correct diagnosis. Therefore, further clinical reports of these rare tumors are essential for raising awareness about the diagnostic process and treatment of such rare cases.

## Case presentation

**Figure 1:**
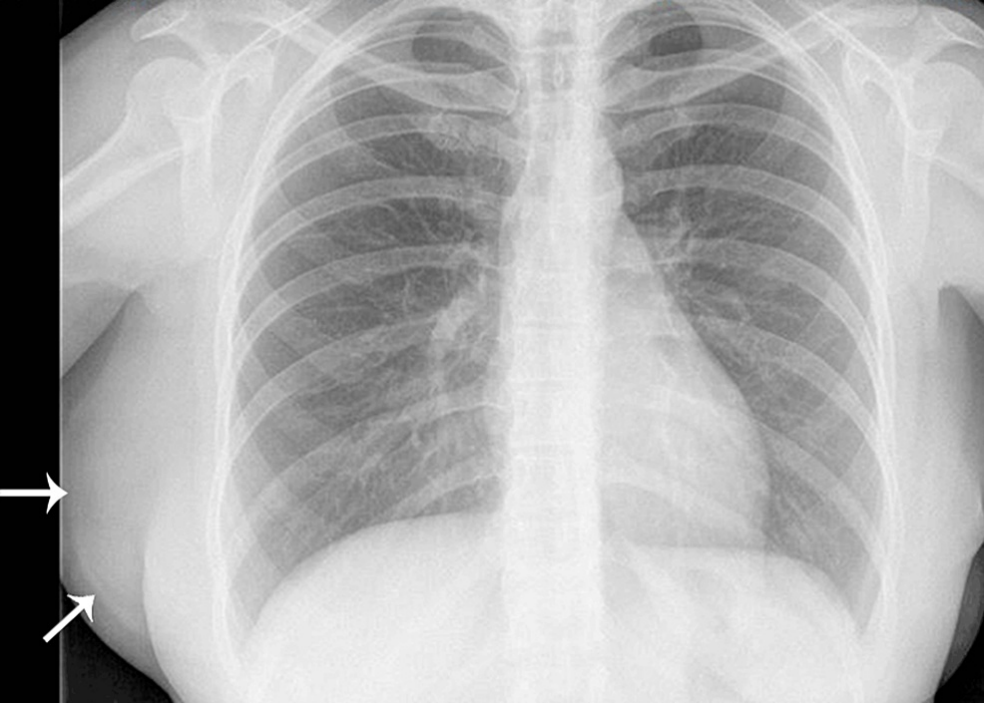
Chest roentgenography presented a clearly defined radiolucent homogenous mass in the the right lateral chest wall, with the same density as surrounding adipose tissue (arrows).

A 38-year-old woman presented with a history of pain and a progressively enlarging mass in her right lateral chest wall. She first noticed the mass 10-15 years ago following a traumatic incident involving a fall onto her right side. Initially, the mass remained stable in size, approximately 5-6 cm, but began enlarging during pregnancy in 2018. Subsequently, it continued growing, causing pain, distress, and functional impairment. Physical examination revealed a large, soft tissue mass measuring 15x5x5 cm in the right lateral chest wall region. The mass appeared homogeneous and soft on palpation. Laboratory tests, encompassing assessments such as white blood cell count, hematocrit, electrolyte levels, and liver function, yielded results within normal ranges. A radiographic evaluation was appointed. The x-ray reviewed a sizeable homogenous mass in the region of the right lateral chest wall, with the same density as surrounding adipose tissue (Figure 1).

**Figure 2:**
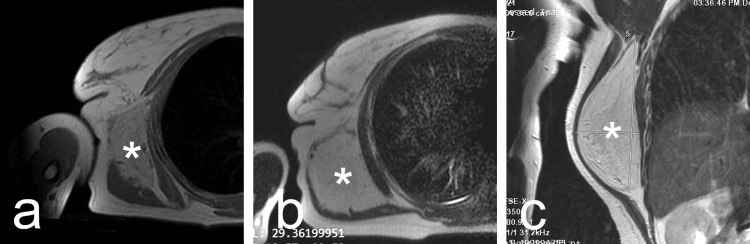
(a-c) Magnetic resonance imaging presented a well-defined, round mass between the serratus anterior and latissimus dorsi muscles with high signal intensity usually slightly less than that of the subcutaneous fat (asterisks).

A following magnetic resonance imaging (MRI) demonstrated a well-defined, round mass between the serratus anterior and latissimus dorsi muscles, measuring 166.5 mm in length and 69 mm in width, with a high signal intensity usually slightly less than that of the subcutaneous fat (Figure 2a-2c).

The radiological examination did not provide enough information for a precise diagnosis, so a provisional diagnosis of an adipose tissue tumor was made. Lipoma, liposarcoma, fibroma, desmoid tumor, or sarcoma were all considered differential diagnoses.

**Figure 3:**
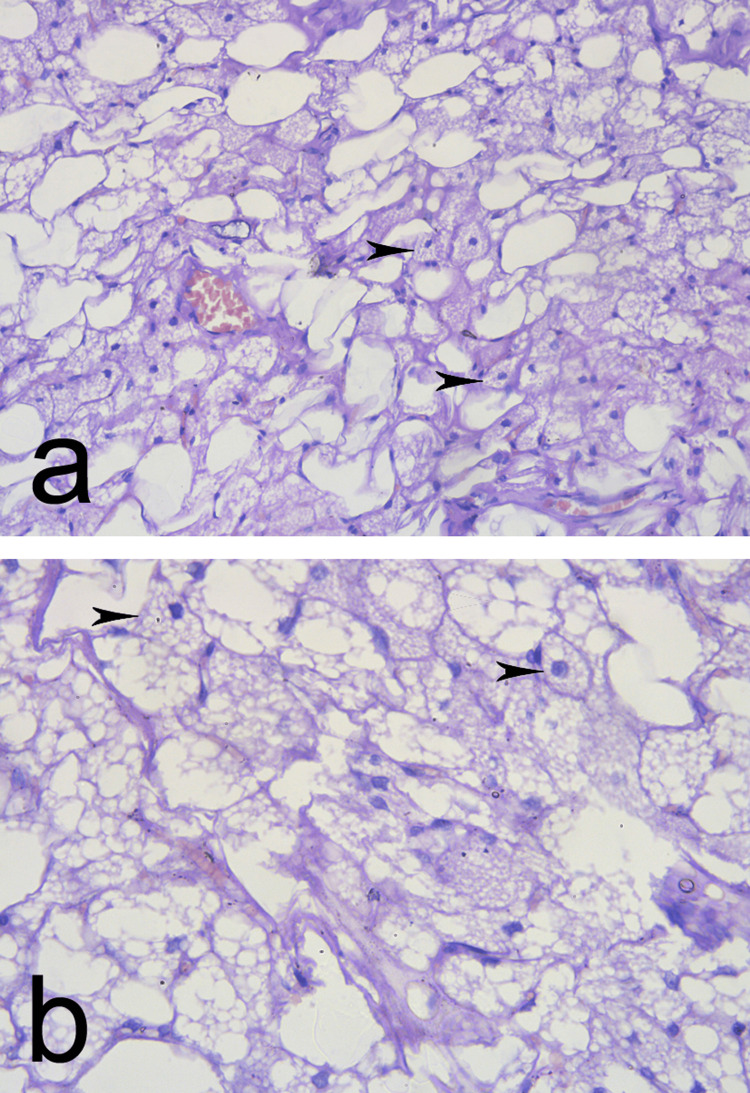
(a, b) Microscopic appearance of the tumor presented by small oval cells with a central nucleus and an abundance of smaller lipid droplets with acidophilic granular cytoplasm (arrowheads). (a) (magnification x 200), (b) (magnification x 400).

An ultrasound-guided thin needle aspiration biopsy was performed, which revealed small spherical cells with a central nucleus and an abundance of smaller lipid droplets with acidophilic granular cytoplasm. These cells were situated in lobules separated by fibrous septs, all characteristics of brown adipose tissue. No evidence of atypia, necrosis, or mitotic activity was observed, and all features were consistent with the typical histopathological appearance of hibernoma (Figures 3a, 3b).

**Figure 4:**
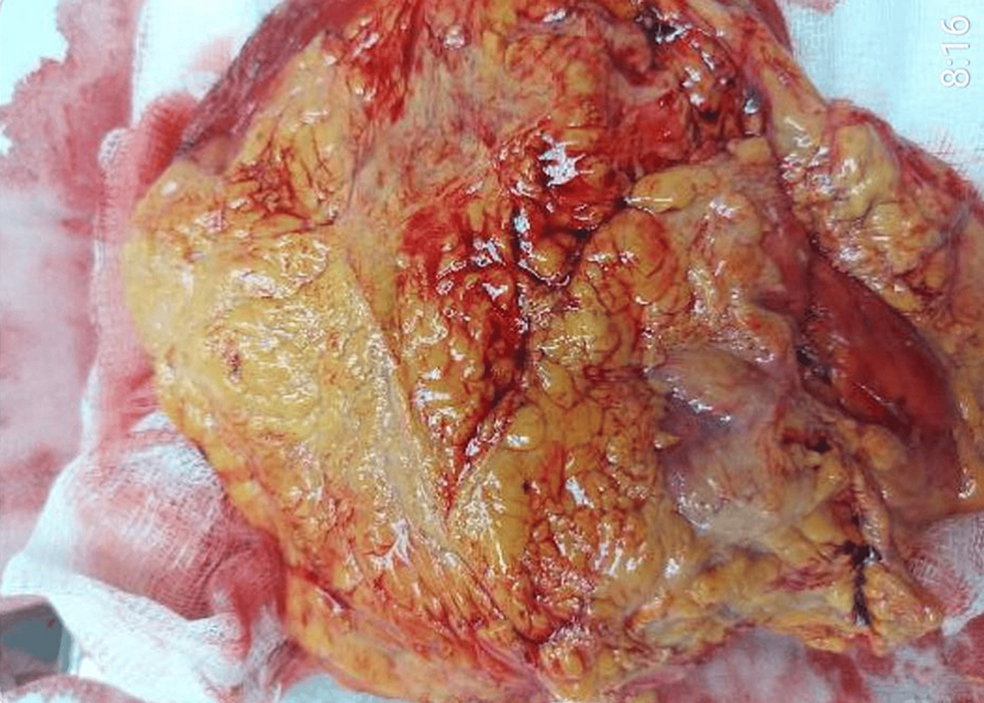
Macroscopical view of the excised hibernoma presented as a fatty, hypervascular lesion that are grossly similar to a lipoma.

Subsequent surgical excision of the hibernoma was performed. A vertical skin incision was made over the middle of the mass, followed by a thorough layer by layers of tissue dissection. The mass between the latissimus dorsi and serratus anterior muscles was identified and preserved in a well-formed fibrous capsule. Total excision of the mass was achieved, followed by hemostasis, and the surgical wound was closed layer by layer, as usual. Macroscopically, the tumor appeared to be a round adipose mass measuring 17x7x6 cm (Figure 4).

The patient was discharged three days after the operation without any postoperative complications. The patient achieved complete recovery without evidence of recurrence during two years of follow-up.

## Discussion

Hibernoma is a rare variant of benign adipose tissue lesions [8,9]. It typically exhibits minimal symptoms and slow progression due to its benign nature [3]. Symptoms usually only manifest when the tumor grows significantly enough to exert pressure on surrounding structures. These tumors are predominantly found in the thigh region during adulthood (30%) of cases, followed by the shoulder (12%), back (10%), neck (9%), chest (6%), arm (6%), and abdominal cavity/retroperitoneum (5%) [2].

Hibernomas commonly appear as fatty, highly vascularized growths, resembling lipomas/liposarcomas in their gross appearance. These lesions are typically well-circumscribed and movable, with colors ranging from tan to red-brown, influenced by the lipid content. Unlike adult adipocytes, which feature nuclei positioned towards the edge of the cell with clear cytoplasm, hibernomas exhibit centrally located nuclei and cytoplasm characterized by multiple vacuoles and a granular eosinophilic appearance [10]. The characteristics used to diagnose hibernomas involve identifying a distinct, lobulated mass with a yellow-brown appearance and cellular properties resembling brown fat, such as multiple small vacuoles within the cytoplasm and granular eosinophilic cytoplasm [11,12]. Hibernoma cells typically have a polygonal shape and exhibit cytological features considered benign, including small central nuclei, noticeable nucleoli, and few instances of abnormal cell morphology or mitotic figures [11]. Unlike hibernomas, conventional lipomas consist mainly of white fat cells [13]. Histologically, hibernomas are categorized into four subtypes based on varying degrees of cytoplasmic eosinophilia, the presence of myxoid stroma, and spindle cell configuration, with their prevalence influenced by demographic factors [11]. Typical hibernomas represent approximately 82% of cases, followed by the myxoid variant at 9%, often mistaken for liposarcoma due to its myxoid stroma. The lipoma-like subtype, accounting for 7% of cases, features scattered hibernoma cells among multivacuolated mature adipocytes and is frequently confused with liposarcoma. The least common spindle-cell variant at 2% exhibits characteristics of both hibernoma and spindle-cell lipoma [14,15]. Despite their varied histological presentations, all subtypes of hibernoma share a similar prognosis, rendering their classification clinically insignificant for most surgical pathologists [11,15]. Based on the provided histopathological classification, our case meets the criteria for the typical variant.

Diagnostic imaging plays a crucial role in the initial assessment of fatty tumors, with plain X-rays and CT scans offering insights into the tumor's fat density [16]. Ultrasonography results may be unclear, but MRI is the gold standard for detailing the tumor's location, size, extent, and characteristics [17]. Hibernomas typically appear as heterogeneous masses with significant contrast enhancement, exhibiting intermediate signal intensity between subcutaneous fat and muscle on CT and MRI scans [18]. Although they contain brown fat, T1- and T2-weighted images show high signal intensity, slightly lower than subcutaneous fat. Fat suppression sequences sometimes show incomplete suppression due to lipid nature and quantity [17,18].

Nevertheless, an accurate diagnosis of hibernoma solely based on radiological examination is nearly impossible. Therefore, a biopsy is usually performed, as most commonly, an ultrasound-guided thin needle aspirational biopsy is preferred [19]. However, it should be noted that the biopsy poses a risk of hemorrhage due to the highly vascularized nature of hibernomas. Moreover, often, the results of biopsies are inconclusive [11].

A definitive solution to the diagnostic challenge posed by hibernomas lies in wide local excision with negative margins, allowing for accurate differentiation from other lipomatous lesions and reducing the risk of recurrence [20]. In a retrospective analysis spanning two decades at a single center, 19 cases of hibernomas were reviewed, with only 7% of cases posing diagnostic challenges in radiology reports where other lesions like atypical lipomatous tumors or well-differentiated liposarcomas could not be ruled out. Surgical excision was performed in all such cases, resulting in no distant metastasis or recurrence, except for one case with positive margins leading to recurrence rather than a true relapse [14].

## Conclusions

The current study presents a rare case of large lateral chest wall hibernoma. Even though hibernomas are benign tumors, their rarity and slow growth complicate the diagnosis. Furthermore, more often than not, imaging methods such as x-rays and CT are not informative enough, and an MRI is advised for lesions bigger than 5 cm. Moreover, a biopsy is preferred prior to surgical treatment. Therefore, cases like the present one are essential to raise awareness among surgeons and radiologists about this rare condition and to outline the proper management and treatment for optimal results.
